# HIF-1α activation in myeloid cells accelerates dextran sodium sulfate-induced colitis progression in mice

**DOI:** 10.1242/dmm.033241

**Published:** 2018-07-30

**Authors:** Young-Eun Kim, Minji Lee, Hyejung Gu, Jeongwoo Kim, Seongju Jeong, Sujin Yeo, You Jeong Lee, Sin-Hyeog Im, Young-Chul Sung, Hak Jae Kim, Irving L. Weissman, G-One Ahn

**Affiliations:** 1 Division of Integrative Biosciences and Biotechnology, Pohang University of Science and Technology (POSTECH), 77 Cheong Am-Ro, Nam-Gu, Pohang, Gyeongbuk 37673, South Korea; 2 Academy of Immunology and Microbiology, Institute for Basic Science, Pohang, Gyeongbuk 37673, South Korea; 3 Department of Life Sciences, POSTECH, 77 Cheong Am-Ro, Nam-Gu, Pohang, Gyeongbuk 37673, South Korea; 4 Department of Radiation Oncology, Seoul National University College of Medicine, Seoul 03080, South Korea; 5 Cancer Research Institute, Seoul National University College of Medicine, Seoul 03080, South Korea; 6 Stem Cell Institute and Regenerative Medicine, Stanford University School of Medicine, 265 Campus Drive, Stanford, CA 94305, USA

**Keywords:** Hypoxia-inducible factor, Myeloid cells, Dextran sodium sulfate, Colitis

## Abstract

Inflammatory bowel disease (IBD) is a chronic inflammatory disease, in which the intestinal epithelium loses its barrier function. Given the existence of the oxygen gradient in the intestinal epithelium and that inflammation further contributes to the tissue hypoxia, we investigated the role of hypoxia-inducible factor (HIF), a transcription factor activated under hypoxic conditions in myeloid cells, in the progression of IBD. To do this, we utilized myeloid-specific knockout (KO) mice targeting HIF pathways, created by a Cre-loxP system with human MRP8 (hMRP8), an intracellular calcium-binding protein, as the myeloid promoter. By feeding 5% dextran sodium sulfate (DSS) to hMRP8 von Hippel Lindau (*Vhl*) KO mice, in which HIF-1α and HIF-2α are constitutively activated in myeloid cells, we found that these mice were highly susceptible to DSS-induced colitis, demonstrating greater body weight loss, increased mortality, faster onset of rectal bleeding, shortened colon length, and increased CD11b- or Gr-1-positive myeloid cells in the colon compared with wild-type (WT) mice. These parameters were restored to, if not better than, the WT levels when we examined hMRP8 *Hif-1a* KO mice upon 5% DSS feeding. hMRP8 *Hif-2a* KO mice, on the other hand, exhibited a similar degree of DSS-induced colitis to that of WT mice. Lastly, when DSS was given together with azoxymethane to induce tumorigenesis in the colon, we found that hMRP8 *Hif-1a* KO mice exhibited comparable levels of colorectal tumors to those of WT mice, indicating that HIF-1α in myeloid cells is dispensable for tumorigenesis. Collectively, our results suggest that HIF-1α activation in myeloid cells critically regulates IBD progression.

## Abstract (precis)

**Summary:** We challenged myeloid-specific knockout mice targeting the hypoxia-inducible factor (HIF) pathway to dextran sodium sulfate-induced colitis, demonstrating that HIF-1α, but not HIF-2α, activation in myeloid cells regulates colitis severity in mice.

## INTRODUCTION

The gastrointestinal epithelium, located between the anaerobic lumen and the well-vascularized subepithelial mucosa, functions as a protective barrier against luminal antigens (Tambuwala et al., 2010). It has been previously shown, by noninvasive electron paramagnetic resonance imaging, that the small intestine and colon are quite hypoxic, with a partial pressure of oxygen of 11 and 3 mm Hg, respectively (He et al., 1999). Furthermore, within the intestine itself, there is a spectrum of the oxygen gradient, such that the luminal sides (such as villi) are more hypoxic than the basal sides (crypts and submucosa) of the epithelium, as demonstrated with 2-nitroimidazole dye, a hypoxic probe (Glover et al., 2016). Studies have also reported that changes in oxygenation can lead to an altered composition of the gut microbiota in mice (Albenberg et al., 2014), and that the microbiota further contributes to tissue hypoxia by secreting a metabolic product, short-chain fatty acid butyrate (Kelly et al., 2015). These studies thus indicate that oxygen sensing is crucial for homeostasis of the gastrointestinal epithelium.

Cells adapt to a hypoxic condition by activating heterodimeric transcription factors, including hypoxia-inducible factor (HIF), which can regulate cellular survival, metabolism and angiogenesis (Semenza, 2001). HIF is composed of an oxygen-sensitive HIF-1α/2α/3α subunit and an oxygen-insensitive and constitutively expressed HIF-1β subunit (Glover and Colgan, 2011). Under normoxic conditions, HIF-1α/2α/3α is hydroxylated by prolyl hydroxylases (PHDs), followed by a rapid proteasomal degradation mediated by von Hippel-Lindau (VHL) E3 ubiquitin ligase complex (Semenza, 2001). However, under hypoxic conditions, PHDs are inhibited, owing to insufficient oxygen substrate availability, leading to stabilization of HIF-1α/2α/3α, which allows its binding with HIF-1β, enabling it to act as a transcription factor in the nucleus (Semenza, 2001).

HIF in epithelial cells is known to be crucial for providing barrier protection. Studies using epithelial-specific knockout (KO) mice targeting HIF pathways have demonstrated that *Hif-1a* deficiency in fatty acid-binding protein-expressing colonic epithelium results in significantly impaired barrier function through decreased expression of protective genes, including multidrug resistance gene-1, intestinal trefoil factor and *Cd73* (also known as *Nt5e*), in 2, 4, 6-trinitrobenzene sulfonic acid (TNBS)-induced colitis (Karhausen et al., 2004), a mouse colitis model in which T-cell-mediated, Th1-driven immune responses are involved (Wirtz et al., 2007). On the other hand, Shah et al. (2008) have shown that constitutive upregulation of HIF in epithelial cells by targeted disruption of *Vhl* in villin-positive epithelial cells leads to exacerbated colitis in mice through increased expression of macrophage migration inhibitory factor, an HIF-target gene in a dextran sodium sulfate (DSS)-induced colitis model, in which immune responses secondary to disruption of the epithelial barrier prevail (Chassaing et al., 2014). Other studies have demonstrated that HIF is required for barrier protection (Kelly et al., 2015) and that administration of dimethyloxalylglycine (DMOG), a proline hydroxylase inhibitor, to stabilize HIF exerts a significant protective effect against DSS-induced colitis by preventing tumor necrosis factor-α (TNF-α; also known as TNF)-induced epithelial apoptosis (Cummins et al., 2008; Hindryckx et al., 2010). These studies suggest a highly complex role of HIF in epithelial cells during inflammatory bowel disease (IBD) progression.

It is well established that IBD is characterized by the dysregulated immune responses to microbiota in the intestinal mucosa (Sun et al., 2017), and that various populations of immune cells critically modulate the disease progression. Clinical studies have shown that IBD patients have increased regulatory T cells (Makita et al., 2004), CD11b (also known as Itgam) and Gr-1 (also known as Ly6g) double-positive myeloid-derived suppressor cells (Haile et al., 2008), and macrophage infiltration (Mahida, 1993). Myeloid cells, including macrophages and dendritic cells, form a central part of the functional mucosal barrier of the intestine (Cader and Kaser, 2013) by promoting generation of regulatory T cells (Scott et al., 2011). Niess et al. (2005) have demonstrated that a chemokine receptor, CX3CR1, in macrophages and dendritic cells in the lamina propria regulates the severity of IBD, partly through transepithelial dendrite formation, which can lead to an appropriate translocation of commensal bacteria to the lymph node (Medina-Contreras et al., 2011). A more recent study by Campbell et al. (2014) has suggested that NADPH oxidase activities in neutrophils are crucial for resolving IBD. Interestingly, some of the cellular functions have been shown to be altered in or during IBD, such that macrophages isolated from IBD patients are impaired in aldehyde dehydrogenase activities, which are required for producing retinoic acid promoting T and B cell homing (Magnusson et al., 2016).

Because aldehyde dehydrogenase (Shiraishi et al., 2017), CX3CR1 (Zhao et al., 2012) and NADPH oxidase (Diebold et al., 2012) are all HIF downstream targets, the above studies thus suggest that HIF in myeloid cells could be an essential regulator for IBD progression. Indeed, a recent study has demonstrated that mice with HIF-1α deficiency in CD11c (also known as Itgax)-expressing dendritic cells are more susceptible to DSS-induced colitis by impaired activation of regulatory T cells (Flück et al., 2016). However, it is still poorly understood how HIF in myeloid cells regulates IBD. In this study, we investigated a role of HIF in myeloid cells in a DSS-induced IBD model by using a novel strain of myeloid-specific KO mice targeting HIF pathways with human MRP8 (hMRP8) as the myeloid promoter. Myeloid-related protein 8 (MRP8), also known as S100A8, is an intracellular calcium-binding protein, and its expression as a heterodimer complex with other S100 proteins (S100A8/S100A9) has been reported to be a clinically useful biomarker in the sera (Cayatte et al., 2012) and intestinal tissues (Foell et al., 2008) of IBD patients. We hereby report that HIF-1α in myeloid cells critically regulates the susceptibility towards DSS-induced colitis, indicating that HIF-1α in myeloid cells could become a novel therapeutic target to treat the disease.

## RESULTS

### Increased infiltration of myeloid cells expressing HIF-1α in the colon of mice fed with 5% DSS

**Fig. 1.**
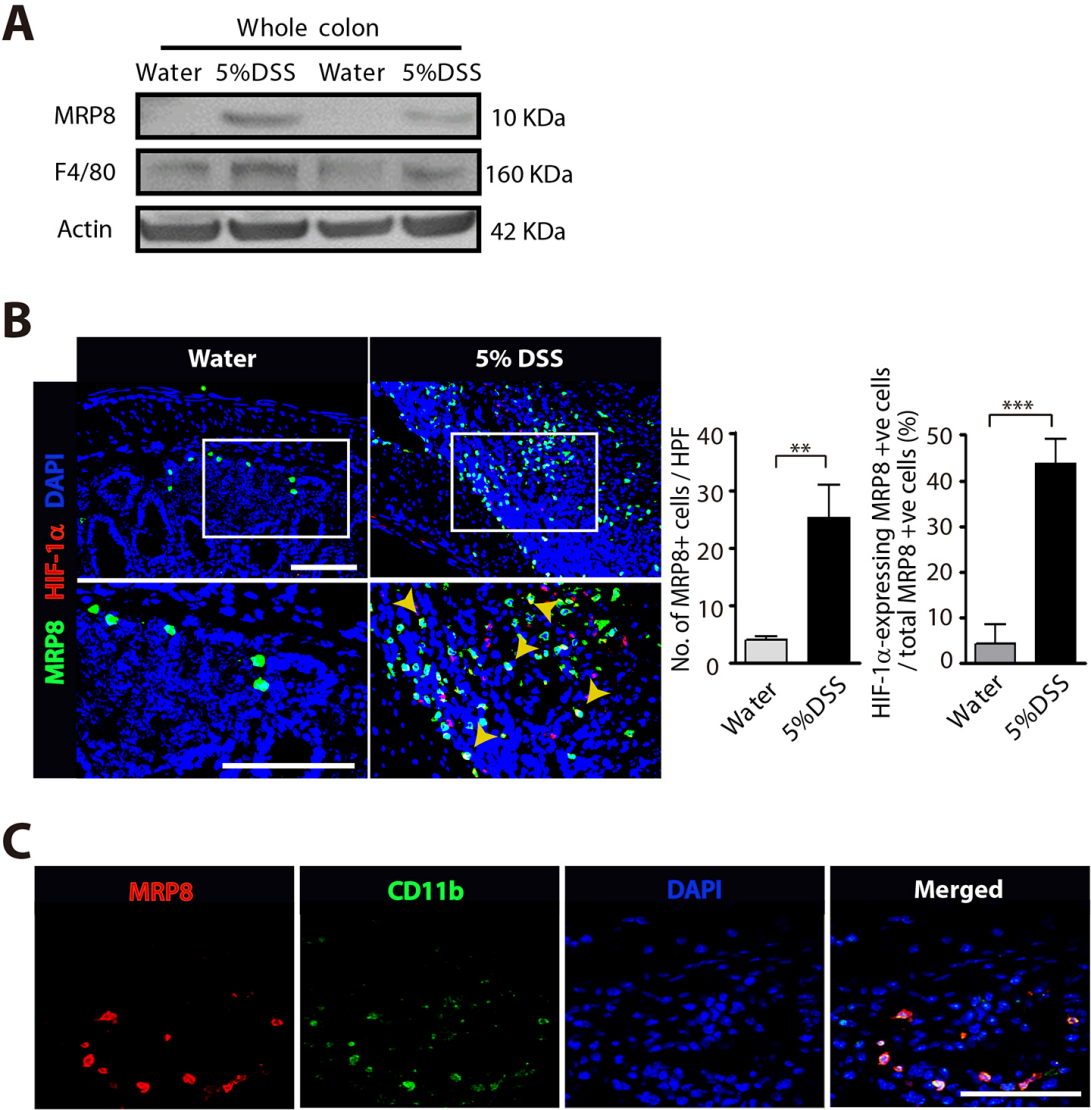
**Increased infiltration of myeloid cells expressing HIF-1α in DSS-induced colitis.** (A) Western blot analysis of MRP8, F4/80 or actin using the whole colon tissue lysate from mice fed with water or 5% DSS for 4 days. (B) Images of the colon of mice fed with water or 5% DSS, immunostained for myeloid cells using anti-MRP8 (green) or anti-HIF-1α (red) antibodies. Nuclei were counterstained with DAPI. White rectangles indicate the areas magnified in the images shown below. Yellow arrowheads indicate cells with colocalization of MRP8 and HIF-1α. Quantification of MRP8-positive cells and percentage of those expressing HIF-1α are shown as bar graphs on the right-hand side. Data are mean±s.e.m. for at least three independent fields examined per mouse (*n*≥3 per group). **P<0.01 and ***P<0.001, assessed by Student's *t*-test. HPF, high-powered field. (C) Images of the colon of mice fed with 5% DSS, immunostained for MRP8 (red) and CD11b (green). DAPI-stained nuclei are shown in blue and a merged image is also shown. Scale bars: 100 μm.

We first examined myeloid cell infiltration in DSS-induced colitis by western blot analysis using the whole colon lysate. We observed that 5% DSS feeding to wild-type (WT) mice resulted in a significant increase in the expression of MPR8 and F4/80 (also known as Adgre1) (Fig. 1A), markers commonly used to detect myeloid cells (Lagasse and Weissman, 1992; Mowat and Bain, 2011), indicating that myeloid cell infiltration is increased in DSS-induced colitis. By performing immunostaining, we were able to see that MRP8-positive myeloid cells were significantly increased in the colon of mice fed with 5% DSS (Fig. 1B), and these cells strongly expressed HIF-1α protein (Fig. 1B). To further verify that MRP8 is a myeloid cell marker, we stained the colon of mice fed with 5% DSS and found that MRP8 was highly colocalized with CD11b in myeloid cells (Fig. 1C).

### The hMRP8 promoter targets Gr-1-positive myeloid cells in the colon

**Fig. 2.**
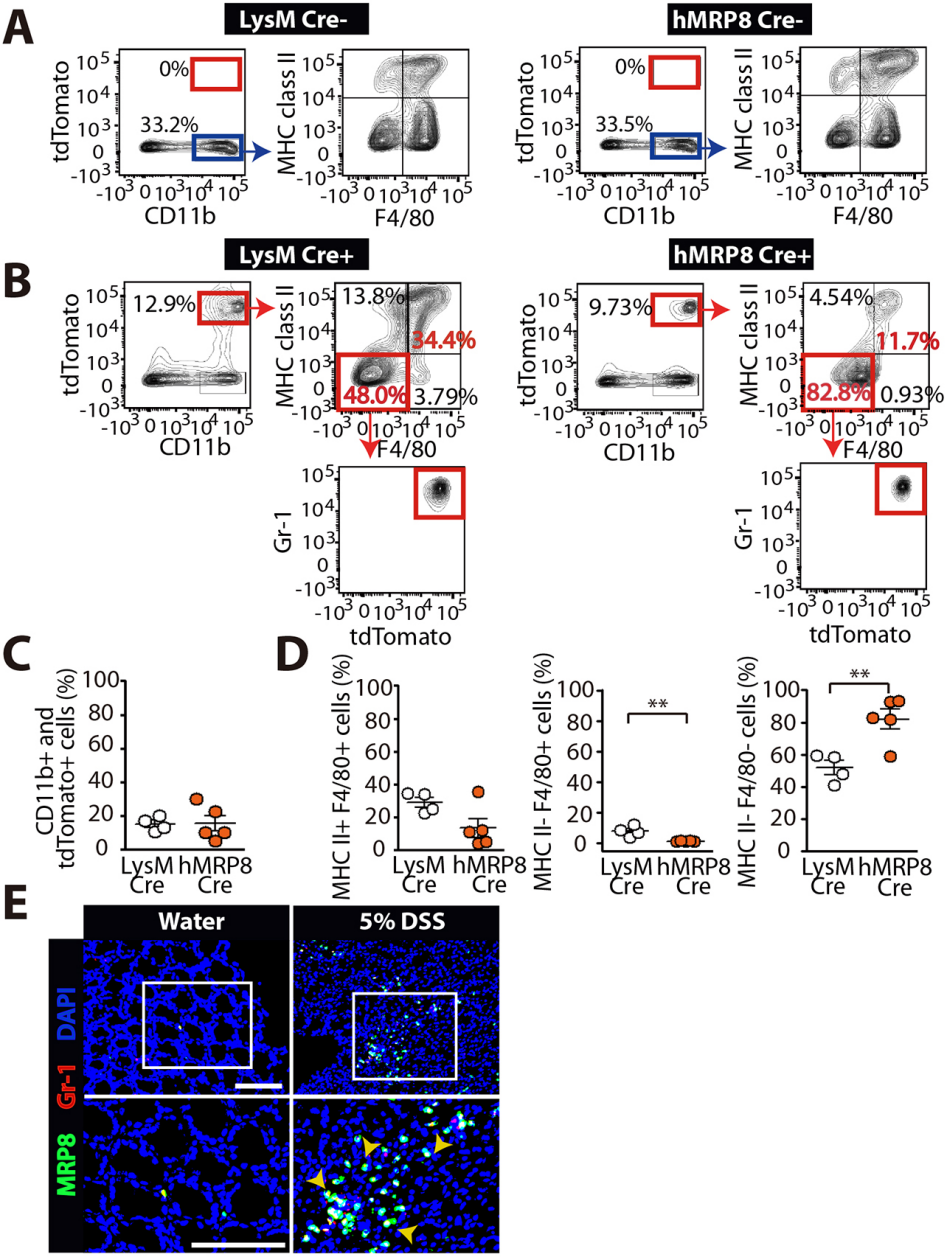
**The hMRP8 myeloid promoter targets Gr-1-positive myeloid cells in the colon.** (A) FACS plots searching for tdTomato-positive populations of cells (red boxes) in the colon of offspring mice not bearing the *Cre* transgene from breeders of LysM Cre (left) or hMRP8 Cre (right) with tdTomato mice. These mice were fed with 5% DSS for 4 days prior to FACS analyses. Note that tdTomato-negative and CD11b-positive myeloid populations (blue boxes) demonstrate similar immunophenotype for MHC class II and F4/80. (B) FACS analyses in the colon of offspring mice bearing the *Cre* transgene that had been fed with 5% DSS. (C) Quantification of tdTomato-positive cells that were also CD11b-positive colonic myeloid cells (red boxes in B) for LysM (*n*=4 mice) and hMRP8 (*n*=5 mice) promoters. (D) Frequencies of different myeloid cell populations (red boxes in B) among the tdTomato and CD11b double-positive population in C. **P<0.01, determined by Student's *t*-test. Data are mean±s.e.m. (E) Images of the colon of mice fed with water or 5% DSS, immunostained for MRP8 (green) and Gr-1 (red). DAPI-stained nuclei are shown in blue. White boxes indicate the areas magnified in the images shown below. Yellow arrowheads indicate cells with MRP8 and Gr-1 colocalization. Scale bars: 100 μm.

To determine a role of HIF in myeloid cells in DSS-induced colitis, we first examined myeloid cell populations targeted by the hMRP8 promoter. We also examined lysozyme M (LysM), another well-known myeloid promoter (Cramer et al., 2003) in parallel. To do this, we cross-bred hMRP8 Cre or LysM Cre mice with ROSA-CAG-LSL-tdTomato-WPRE reporter mice, in which a loxP-flanked STOP cassette is present in a CAG-promoter-driven tandem dimer (td)Tomato fluorescence reporter construct inserted into the *Rosa26* locus (hereafter denoted as tdTomato), and fed the offspring mice with 5% DSS prior to fluorescence-activated cell sorting (FACS) analyses. We observed that there were no tdTomato-positive cells detected in the colon of the offspring mice not bearing the *Cre* recombinase transgene, regardless of whether it was targeted by the LysM or hMRP8 promoter (Fig. 2A). In the offspring mice bearing *Cre* recombinase transgenes, we observed that tdTomato-positive cells were present (Fig. 2B), and that CD11b and tdTomato double-positive myeloid cells were ∼15% of the total colonic mononuclear cells for both LysM and hMRP8 promoters (Fig. 2B,C). Of those, ∼30% and ∼15% were MHC class II and F4/80 double-positive ‘colonic macrophages’ for the LysM and hMRP8 promoters, respectively (Fig. 2B,D), although the frequencies of these populations did not differ significantly between the two promoters (Fig. 2D). LysM Cre reporter mice contained significantly higher MHC class II-negative, but F4/80-positive, ‘immature macrophage’ populations in the colon (Fig. 2B,D). However, these cells constituted relatively a minor fraction in the colonic myeloid cells, accounting for less than ∼10% of the total CD11b and tdTomato double-positive mononuclear cells (Fig. 2B,D). Interestingly CD11b and tdTomato double-positive mononuclear cells of hMRP8 Cre reporter mice were mostly (∼80%) negative for both MHC class II and F4/80 (Fig. 2B,D), and these turned out to be Gr-1 positive (Fig. 2B). Indeed we confirmed that in infiltrating MRP8-positive myeloid cells in the colon of DSS-fed mice, MRP8 was highly colocalized with Gr-1 (Fig. 2E).

### Mice deficient for VHL in myeloid cells are highly susceptible to DSS-induced colitis

**Fig. 3.**
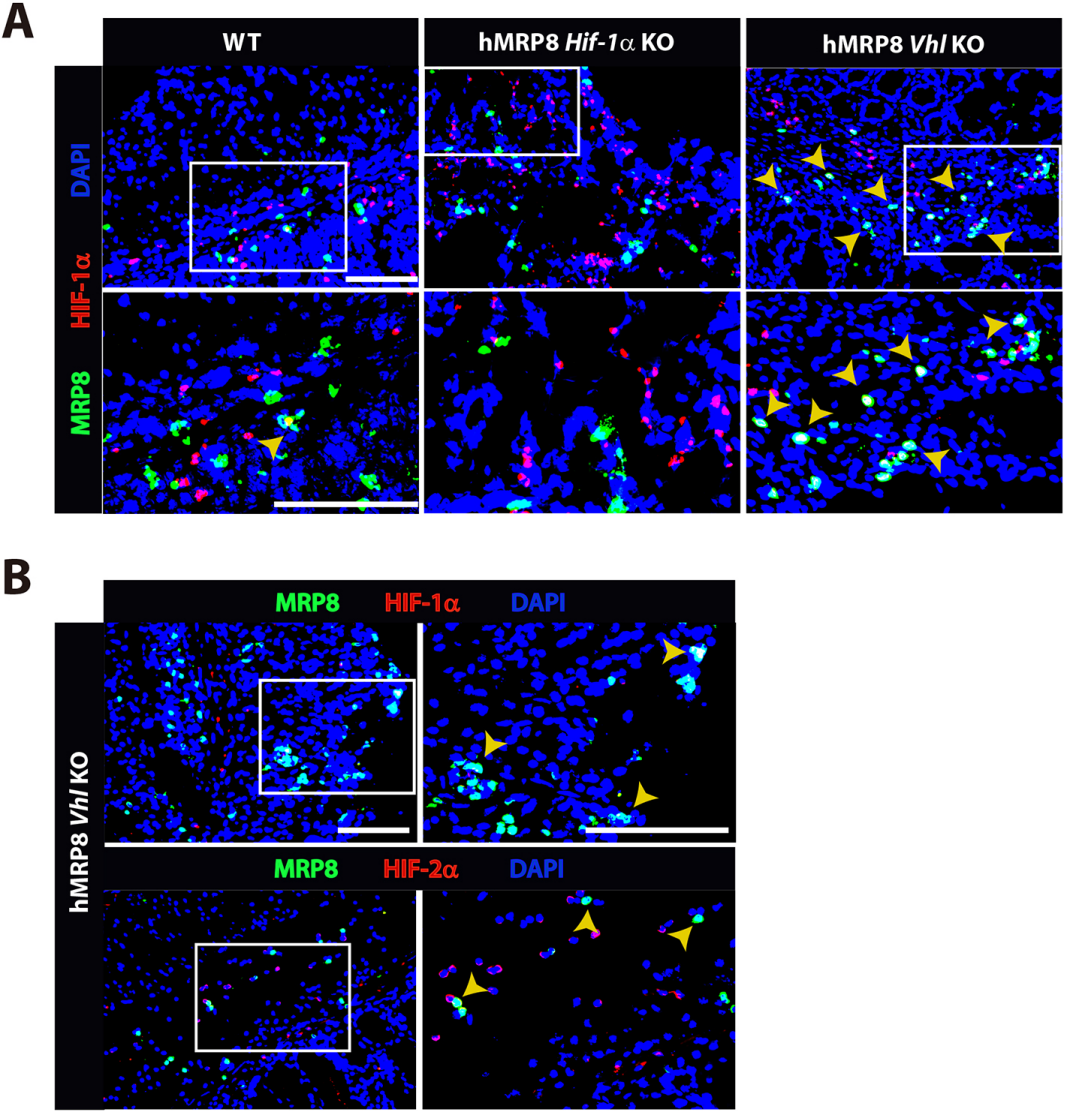
**HIF-1α/2α expression in myeloid-specific KO mice targeting the HIF pathway.** (A) Images of the colon of wild-type (WT), myeloid-specific *Hif-1a* KO (hMRP8 *Hif-1a* KO) or von Hippel Lindau (*Vhl*) KO (hMRP8 *Vhl* KO) mice, immunostained for MRP8 (green) and the DNA-binding regions of *Hif-1a* mRNA (red). Mice were fed with 5% DSS for 4 days prior to immunostaining analyses. Note that there were no MRP8-positive cells that were positive for *Hif-1a* mRNA in hMRP8 *Hif-1a* KO (middle column) mice, but we observed many cells that were double positive for MRP8 and *Hif-1a* mRNA in hMRP8 *Vhl* KO mice (right column). (B) Images of the colon of hMRP8 *Vhl* KO mice fed with 5% DSS as in A, immunostained for MRP8 (green) and HIF-1α (red, upper row) or HIF-2α (red, bottom row). DAPI-stained nuclei are shown in blue. White boxes in A and B indicate the regions magnified in the lower or right images, respectively. Yellow arrowheads in A and B indicate cells positive for both markers. Scale bars: 100 μm.

Next, we examined whether HIF is expressed in myeloid cells in our strain of myeloid-specific KO mice targeting HIF pathways. By utilizing an mRNA probe designed specifically to recognize exon 2 of *Hif-1a*, we observed that there were MRP8-positive myeloid cells expressing *Hif-1a* mRNA in the colon of myeloid-specific *Vhl* KO (hereafter denoted as hMRP8 *Vhl* KO) mice fed with 5% DSS. In contrast, *Hif-1a* mRNA was not detected in MRP8-positive myeloid cells in hMRP8 *Hif-1a* KO mice fed with 5% DSS (Fig. 3A), consistent with the fact that targeted disruption of *Hif-1a* by the loxP site occurs at exon 2 (Ryan et al., 2000). Immunofluorescent staining analyses further demonstrated that MRP8-positive colonic myeloid cells in hMRP8 *Vhl* KO expressed both HIF-1α and HIF-2α protein (Fig. 3B).

**Fig. 4.**
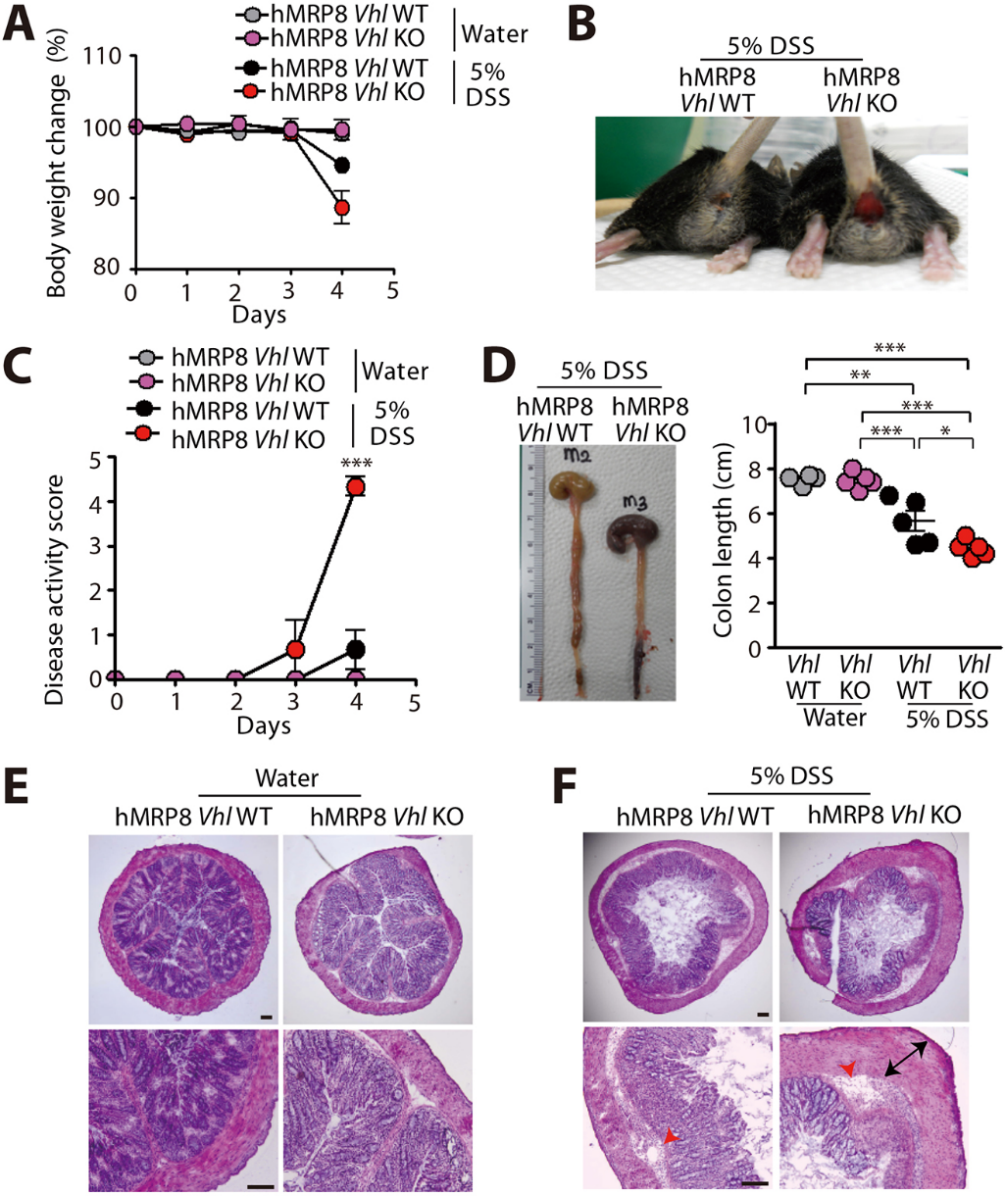
**hMPR8 *Vhl* KO mice are highly susceptible to DSS-induced colitis.** (A-D) Body weight change (A), a photograph demonstrating rectal bleeding (B), disease activity score (C) and colon length (D) of hMPR8 *Vhl* WT or KO mice fed with water (*n*=4 for hMPR8 *Vhl* WT mice; *n*=5 mice for hMPR8 *Vhl* KO mice) or 5% DSS (*n*=5 for hMPR8 *Vhl* WT mice; *n*=5 mice for hMPR8 *Vhl* KO mice) for 4 days. Data in A, C and D are mean±s.e.m. *P<0.05, **P<0.01 and ***P<0.001, determined by two-way (C) or one-way (D) ANOVA. (E,F) Hematoxylin and Eosin (H&E) staining of the colon is also shown for mice fed with water (E) or 5% DSS (F). Red arrowheads and black arrows in F indicate areas of vacuolar hydropic degeneration and thickened muscularis mucosa, respectively. Scale bars: 100 μm.

**Fig. 5.**
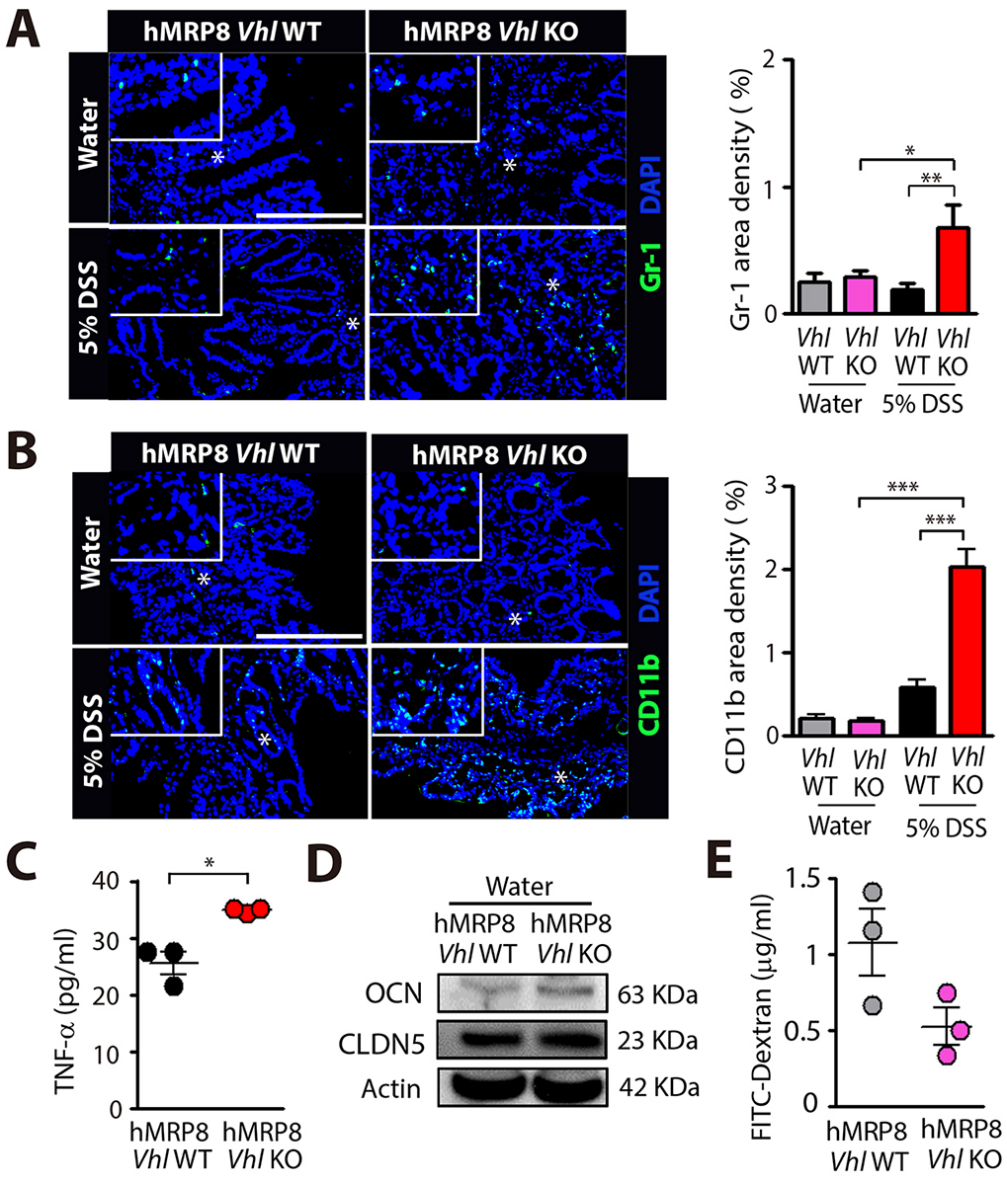
**Increased infiltration of CD11b- and Gr-1-positive myeloid cells secreting TNF-α in hMRP8 *Vhl* KO mice fed with 5% DSS.** (A,B) Images of the colon of hMRP8 WT or *Vhl* KO mice fed with 5% DSS for 4 days, immunostained for Gr-1 (A) or CD11b (B), shown in green. White asterisks indicate the areas shown magnified in the insets. Area densities were quantified and shown as bar graphs on the right-hand side. Data are mean±s.e.m. for at least three independent fields examined per mouse (*n*≥3 mice per group). *P<0.05, **P<0.01 and ***P<0.001, determined by one-way ANOVA. Scale bars: 100 μm. (C) TNF-α concentrations measured in the supernatant containing CD11b and Gr-1 double-positive myeloid cells isolated from the colon of hMRP8 WT or *Vhl* KO mice fed with 5% DSS and incubated for 24 h after FACS sorting. Data are mean±s.e.m. for triplicate determinations. *P<0.05, determined by Student's *t*-test. (D) Western blot for occludin (OCN) and claudin 5 (CLDN5) in the whole colon lysate obtained from hMRP8 WT or *Vhl* KO mice fed with water. (E) Plasma FITC fluorescence level at 4 h after a single oral administration of FITC-dextran in hMRP8 WT or *Vhl* KO mice fed with water. Data are mean±s.e.m. for *n*=3 mice per group.

We then fed hMRP8 *Vhl* KO or littermate WT mice with water or 5% DSS. We observed that hMRP8 *Vhl* KO mice fed with 5% DSS exhibited significant body weight loss at day 4 (Fig. 4A), when we had to euthanize these animals. WT mice demonstrated ∼5% of the initial body weight loss at this time (Fig. 4A). During DSS feeding, we also assessed disease activity according to the following arbitrary scale: 1, small changes in body weight; 2, notable body weight loss; 3, bloody stool; 4, rectal bleeding; and 5, rectal bleeding and near morbidity (>15% of the body weight loss). We observed that hMRP8 *Vhl* KO mice fed with 5% DSS exhibited severe rectal bleeding (Fig. 4B) and worsened disease activity scores (Fig. 4C) than WT mice. Furthermore, the colon length was significantly shorter in these hMRP8 *Vhl* KO mice (Fig. 4D). Water-fed hMRP8 *Vhl* KO mice demonstrated no differences in body weight change (Fig. 4A), disease activity scores (Fig. 4C), colon length (Fig. 4D) and histological features of the colon (Fig. 4E) compared with WT mice. Histological analyses revealed that hMRP8 *Vhl* KO mice fed with 5% DSS had increased erosion of mucosa, vacuolar hydropic degeneration of cells, increased leukocyte infiltration and thickening of the muscularis mucosa compared with WT mice (Fig. 4F). Given the above results that the hMRP8 myeloid promoter efficiently marked CD11b and Gr-1 myeloid cells in the colon, we examined these cells in the colon of hMRP8 *Vhl* KO or WT mice by immunostaining. We found that area densities for Gr-1-positive (Fig. 5A) or CD11b-positive (Fig. 5B) myeloid cells were significantly increased in hMRP8 *Vhl* KO mice compared with WT mice when they were fed with 5% DSS. When fed with water, these infiltrating myeloid cells did not differ between hMRP8 *Vhl* KO and WT mice (Fig. 5A,B). We then isolated CD11b and Gr-1 double-positive cells from the colon of animals that had been fed with 5% DSS by FACS and measured TNF-α, a major cytokine secreted by DSS-treatment, thereby regulating IBD progression (Xiao et al., 2016), and a HIF-1 downstream target (Xing et al., 2015). We observed that myeloid cells from DSS-fed hMRP8 *Vhl* KO mice produced significantly higher levels of TNF-α than those from WT mice (Fig. 5C). To exclude the possibility that the increased susceptibility of hMRP8 *Vhl* KO mice to DSS-induced colitis was caused by barrier defects associated with hMRP8 *Vhl* KO, we examined barrier proteins, including occludin (OCN) and claudin 5 (CLDN5), in the colon of mice fed with water, and observed that there were no significant differences in the expression of these proteins between hMRP8 *Vhl* KO and WT mice (Fig. 5D). Furthermore, the plasma fluorescein isothiocyanate (FITC) levels following oral gavage administration of FITC-dextran were also similar in these mice (Fig. 5E), suggesting that the barrier functions were comparable between hMRP8 *Vhl* KO and WT mice. These results thus indicate that hMRP8 *Vhl* KO mice are more susceptible to DSS-induced colitis through increased infiltration of CD11b- and Gr-1-expressing myeloid cells secreting TNF-α.

### hMRP8 *Hif-1a* KO mice, but not hMRP8 *Hif-2a* KO mice, are protected from 5% DSS-induced colitis

**Fig. 6.**
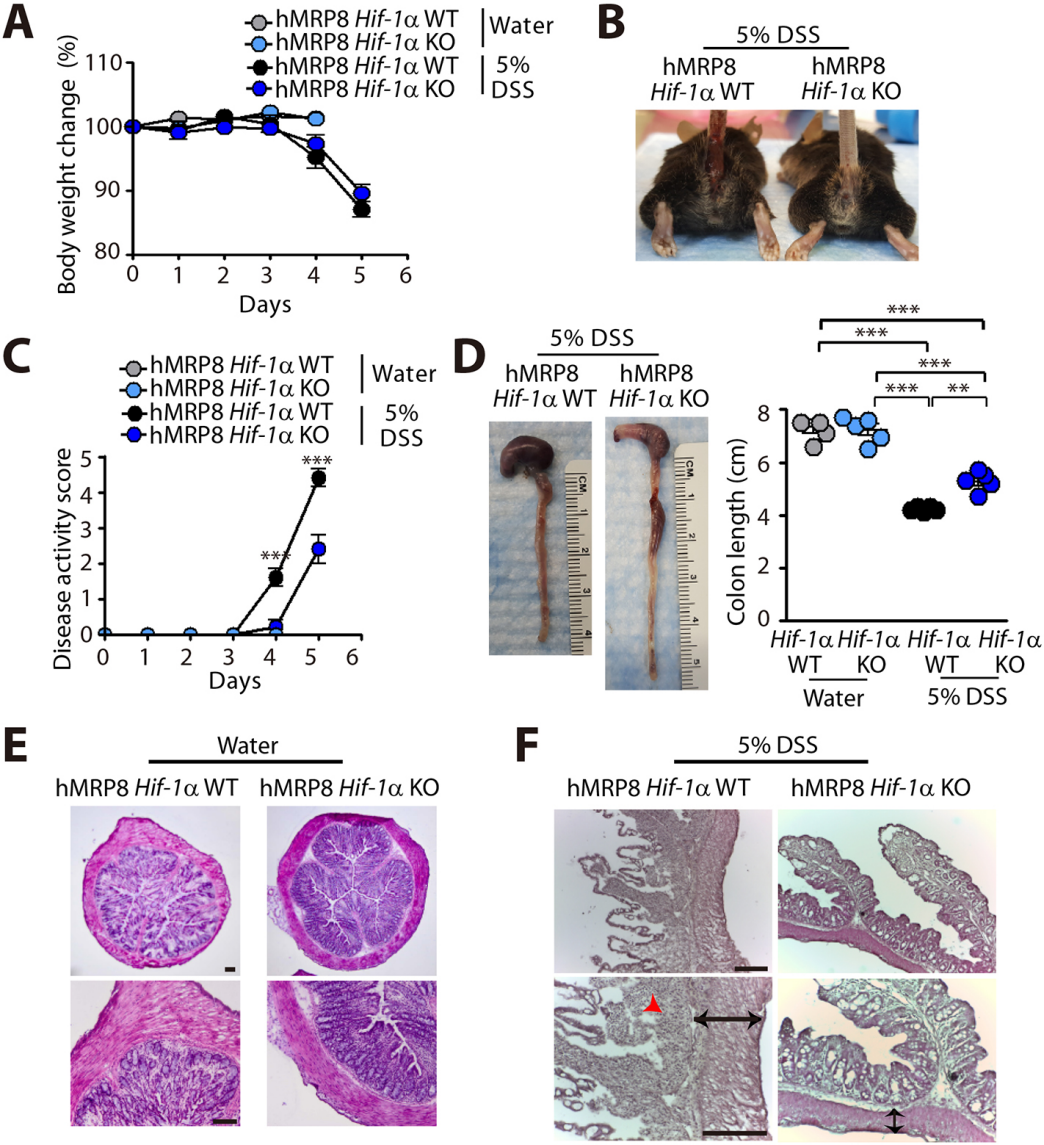
**hMRP8 *Hif-1a* KO mice are protected from DSS-induced colitis.** (A-D) Body weight change (A), a photograph of the rectal area (B), disease activity score (C) and colon length (D) of hMRP8 WT or *Hif-1a* KO mice fed with water for 4 days or 5% DSS for 5 days. Data in A, C and D are mean±s.e.m. for *n*=5 mice per group. **P<0.01 and ***P<0.001, determined by two-way (C) or one-way (D) ANOVA. (E,F) H&E staining of the colon is also shown for mice fed with water (E) or 5% DSS (F). Black arrows in F indicate the thickened muscularis mucosa observed in WT mice; red arrowhead indicates inflammatory cells. Scale bars: 100 μm.

**Fig. 7.**
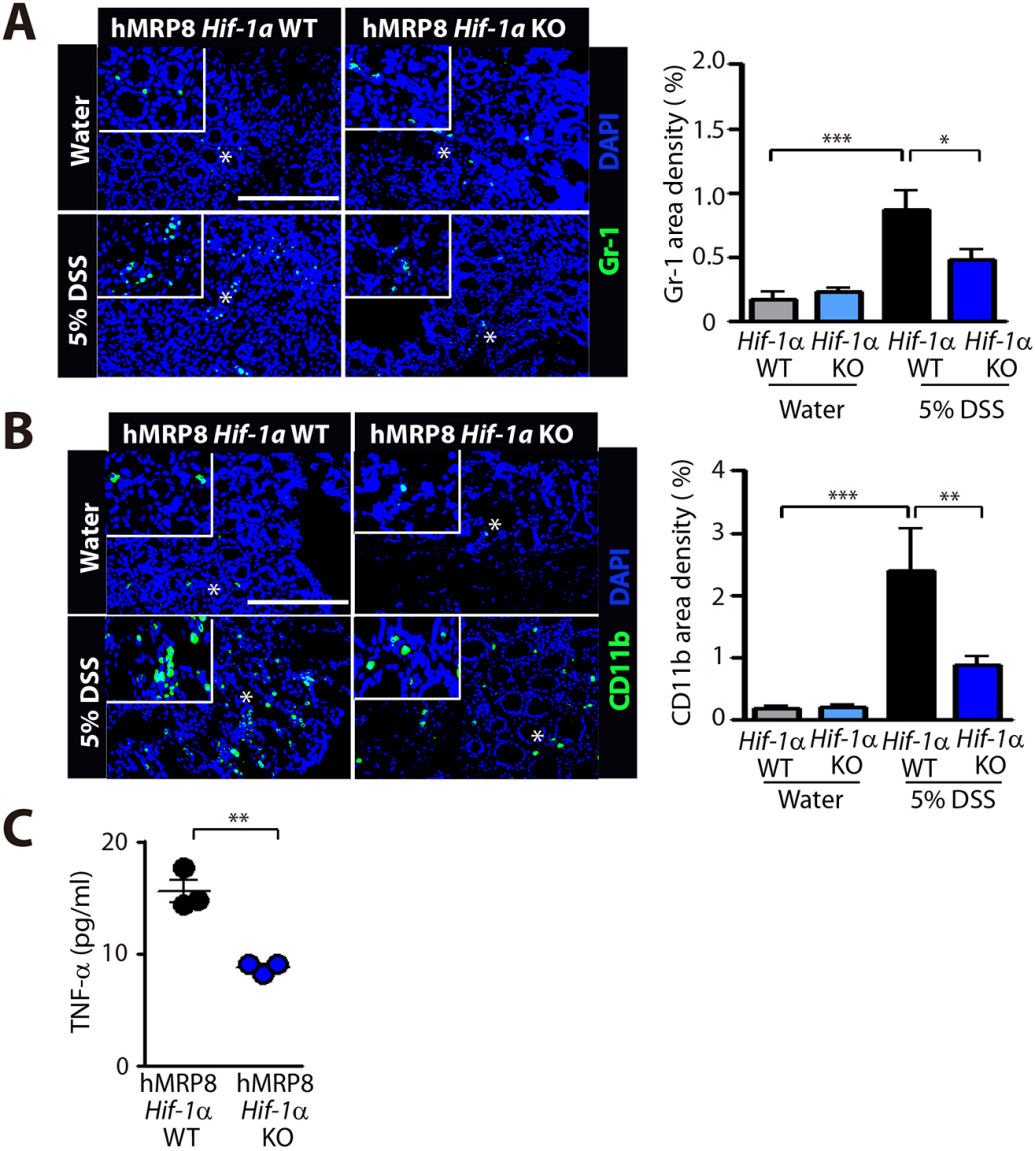
**Decreased infiltration of CD11b- and Gr-1-positive myeloid cells and reduced TNF-α secretion in hMRP8 *Hif-1a* KO mice fed with 5% DSS.** (A,B) Images of the colon of hMRP8 *Hif-1a* WT or *Hif-1a* KO mice fed with water for 4 days or 5% DSS for 5 days, immunostained for Gr-1 (A) or CD11b (B), shown in green. White asterisks indicate the areas shown magnified in the insets. Area densities were quantified and shown as bar graphs on the right-hand side. Data are mean±s.e.m. for at least three independent fields examined per mouse (*n*≥3 mice per group). *P<0.05, **P<0.01 and ***P<0.001, determined by one-way ANOVA. Scale bars: 100 μm. (C) TNF-α concentrations measured in the supernatant containing CD11b and Gr-1 double-positive myeloid cells isolated from the colon of hMRP8 WT or *Hif-1a* KO mice fed with 5% DSS for 5 days and incubated for 24 h after FACS sorting. Data are mean±s.e.m. for triplicate determinations. **P<0.01, determined by Student's *t*-test.

Because VHL loss results in constitutive activation of HIF-1α/2α (Haase, 2006), we wanted to determine whether DSS-induced colitis features that were observed in hMRP8 *Vhl* KO mice would be reversed in myeloid-specific hMRP8 *Hif-1a* KO mice. By feeding 5% DSS, we first observed that there was no significant difference in the body weight loss between hMRP8 *Hif-1a* KO and WT mice, which demonstrated ∼10-15% body weight loss by day 5 (Fig. 6A), when we euthanized the animals. However, hMRP8 *Hif-1a* KO mice fed with 5% DSS demonstrated less severe rectal bleeding (Fig. 6B) and improved disease activity scores (Fig. 6C) compared with WT mice. Upon dissection, we observed that the colon length of hMRP8 *Hif-1a* KO mice was significantly longer than that of WT mice (Fig. 6D), although these were all shortened by DSS administration (Fig. 6D). We further observed that although the mucosal architectures were similar between WT and hMRP8 *Hif-1a* KO mice fed with water (Fig. 6E), hMRP8 *Hif-1a* KO mice fed with 5% DSS had better preserved mucosa, submucosa and muscularis mucosa layers compared with WT mice fed with 5% DSS (Fig. 6F). Immunostaining analyses revealed that Gr-1 (Fig. 7A)- and CD11b (Fig. 7B)-positive myeloid cell infiltration in the colon was significantly reduced in DSS-fed hMRP8 *Hif-1a* KO mice (Fig. 7A,B). Furthermore, we observed significantly reduced TNF-α secretion from FACS-isolated CD11b and Gr-1 double-positive myeloid cells from the colon of hMRP8 *Hif-1a* KO mice fed with 5% DSS, compared with those from the colon of WT mice (Fig. 7C).

**Fig. 8.**
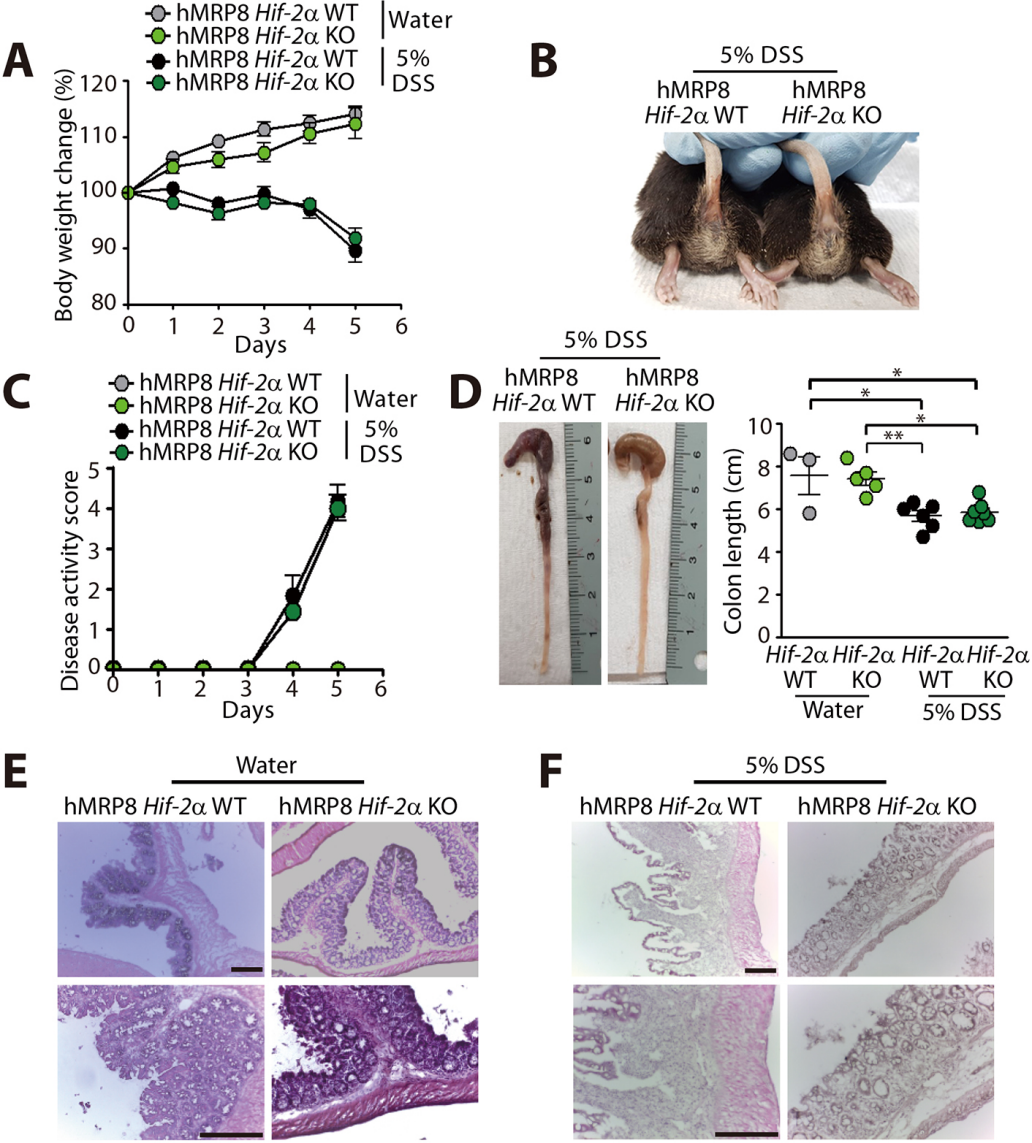
**hMRP8 *Hif-2a* KO mice exhibit a similar extent of DSS-induced colitis compared with WT mice.** (A-D) Body weight change (A), a photograph of rectal bleeding (B), disease activity score (C) and colon length (D) in hMRP8 *Hif-2a* WT or *Hif-2a* KO mice fed with water (*n*=3 for hMRP8 *Hif-2a* WT mice; *n*=5 mice for hMRP8 *Hif-2a* KO mice) or 5% DSS (*n*=6 for hMRP8 *Hif-2a* WT mice; *n*=7 for hMRP8 *Hif-2a* KO mice) for 5 days. Data in A, C and D are mean±s.e.m. *P<0.05 and **P<0.01, determined by one-way ANOVA. (E,F) H&E staining of the colon is also shown for mice fed with water (E) or 5% DSS (F). Scale bars: 100 μm.

**Fig. 9.**
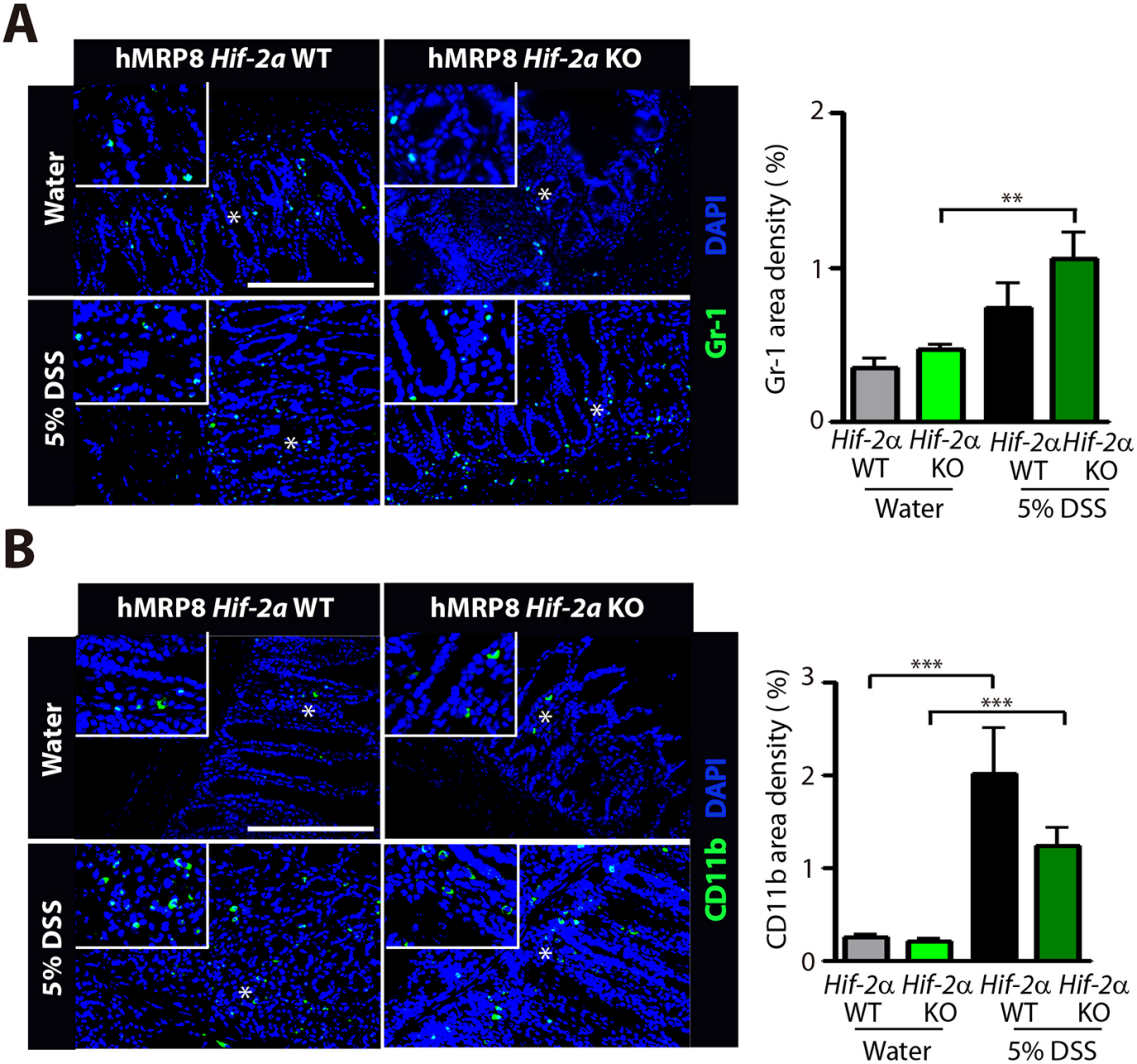
**DSS-fed hMRP8 *Hif-2a* KO mice demonstrate comparable myeloid cell infiltration to the colon.** (A,B) Images of the colon of hMRP8 WT or *Hif-2a* KO mice fed with water or 5% DSS for 5 days, immunostained for Gr-1 (A) or CD11b (B), shown in green. White asterisks indicate the areas shown magnified in the insets. Area densities were quantified and shown as bar graphs on the right-hand side. Data are mean±s.e.m. for at least three independent fields examined per mouse (*n*≥3 mice per group). **P<0.01 and ***P<0.001, determined by one-way ANOVA. Scale bars: 100 μm.

We next examined whether the protection from DSS-induced colitis in hMRP8 *Hif-1a* KO mice would also occur in hMRP8 *Hif-2a* KO mice. We observed that the body weight loss (Fig. 8A), extent of rectal bleeding (Fig. 8B), disease activity scores (Fig. 8C) and colon length shortening (Fig. 8D) were all similar between hMRP8 *Hif-2a* KO and WT mice fed with 5% DSS. Moreover, histological examination revealed similar morphology of the colonic structure between hMRP8 *Hif-2a* KO and WT mice in water (Fig. 8E)- or DSS (Fig. 8F)-fed groups. Immunostaining of Gr-1 (Fig. 9A) and CD11b (Fig. 9B) revealed that these myeloid cells were increased in the colon of 5% DSS-fed animals and the levels did not differ between hMRP8 *Hif-2a* KO and WT mice (Fig. 9A,B). Therefore, the results collectively indicate that hMRP8 *Hif-1a* KO mice, but not hMRP8 *Hif-2a* KO mice, were protected from DSS-induced colitis.

### HIF activation in myeloid cells is dispensable for azoxymethane (AOM)-induced tumorigenesis

**Fig. 10.**
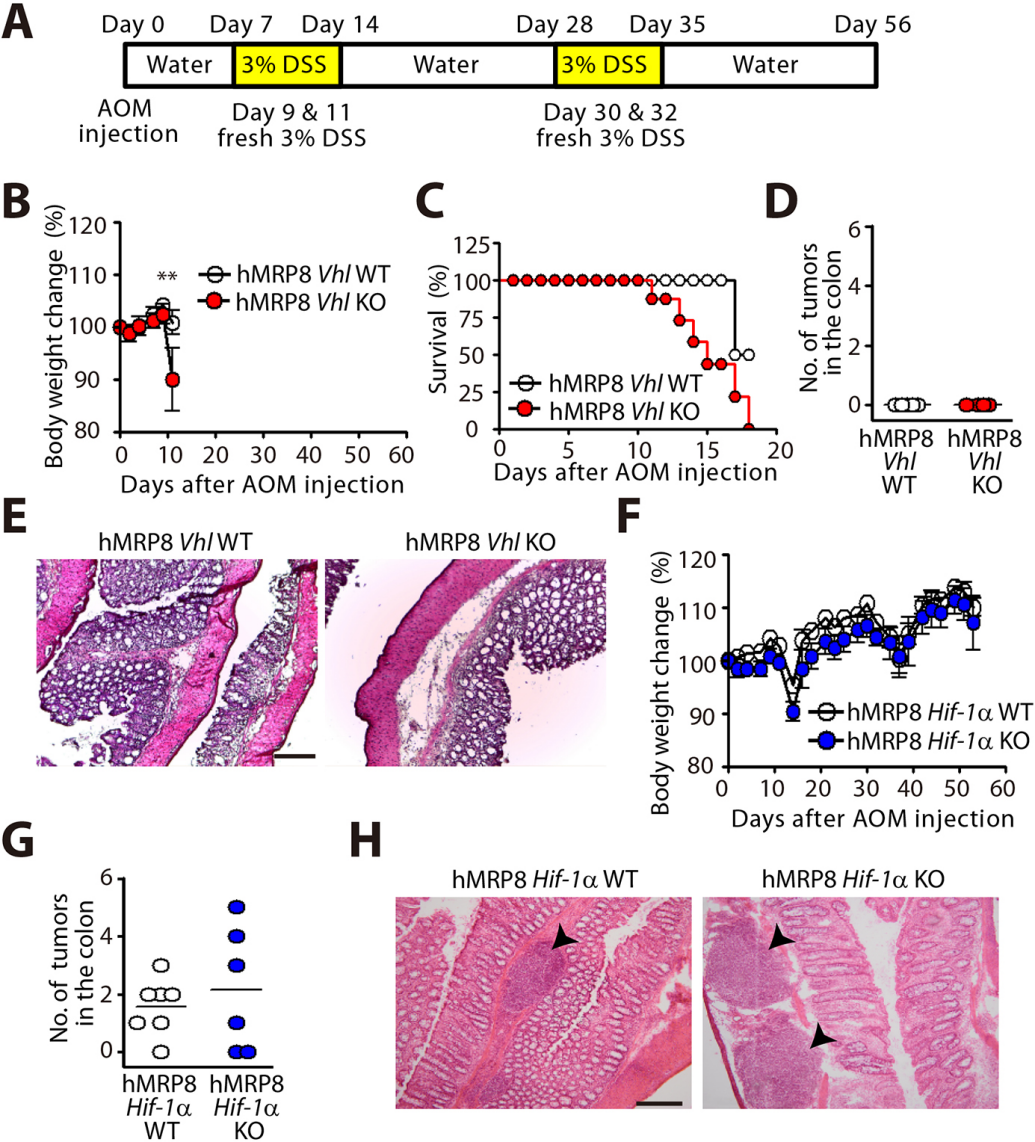
**Myeloid HIF-1α is dispensable for colitis-associated colorectal tumorigenesis.** (A) Scheme of AOM/DSS treatment to induce colitis-associated colorectal cancer. (B) Changes in the body weight in hMRP8 *Vhl* WT (*n*=5) or *Vhl* KO (*n*=4) mice treated as in A. **P<0.01, determined by two-way ANOVA. (C) Kaplan–Meier survival plot of the animals in B. (D) Number of macroscopic tumors in the colon of the animals in B. (E) H&E staining of the colon obtained from the animals in B. (F) Body weight changes in hMRP8 *Hif-1a* WT (*n*=8) or *Hif-1a* KO (*n*=7) mice treated as in A. (G) Number of macroscopic tumors counted per colon from the animals in F. Data in B-D and F are mean±s.e.m. (H) H&E staining of the colon of the animals in F. Black arrowheads indicate the tumor regions. Scale bars: 100 μm.

To determine whether the colitis susceptibility in myeloid-specific KO mice targeting HIF pathways would lead to tumorigenesis, we combined the DSS-feeding protocol with AOM injection to induce colorectal tumors (Fig. 10A). We found that hMRP8 *Vhl* KO mice exhibited a rapid body weight loss (Fig. 10B) followed by sudden mortality (Fig. 10C), indicating that these mice are highly susceptible to inflammation in the colon. At this time, we found no tumors in the colon of either hMRP8 *Vhl* KO or WT mice (Fig. 10D,E). Because the rapid body weight loss and high mortality rate in hMRP8 *Vhl* KO mice treated with AOM and DSS prevented us from investigating the role of HIF in myeloid cells in tumorigenesis, we decided to perform the study in hMRP8 *Hif-1a* KO mice instead. We observed that that body weight changes in hMRP8 *Hif-1a* KO mice injected with AOM followed by DSS administration were similar to those of WT mice (Fig. 10F). Upon examining the colon of these animals at the end of the study, we found tumors in the colon of hMRP8 *Hif-1a* KO mice, and observed that the number of macroscopic tumors (Fig. 10G) or histological characteristics of these tumors, revealing adenoma formed in the submucosal layers (Fig. 10H), were comparable between hMRP8 *Hif-1a* KO and WT mice. These results indicate that although inactivation in HIF-1α in myeloid cells suppressed DSS-induced colitis susceptibility, it was dispensable for AOM-induced tumorigenesis.

## DISCUSSION

Gut epithelial cells are exposed to a steep gradient of oxygen owing to the hypoxic characteristic of the gut lumen (Zeitouni et al., 2016), and an inflammatory condition can lead to even more severe hypoxia (Taylor and Colgan, 2007). HIF is therefore essential in IBD progression and indeed *Hif-1a* and *Hif-2a* overexpression is observed in surgical specimens from ulcerative colitis and Crohn's disease (Giatromanolaki et al., 2003). In particular, HIF-1α expression is observed focally, not only in epithelial cells but also in stromal cells (Giatromanolaki et al., 2003), consistent with our results of HIF-1α expression in MRP8-positive cells (Fig. 1). MRP8, also known as calprotectin or S100A8, is present in the cytosol of myeloid cells and can be secreted extracellularly upon stimulation by IL-1β and TNF-α (Ryckman et al., 2003). It has been reported that MRP8 secretion from colonocytes mirrors fecal excretion of leukocytes (Røseth et al., 1992), and that fecal MRP8 levels closely mirror disease activity, thereby serving as a potential biomarker for IBD screening in adult patients (van Rheenen et al., 2010).

Myeloid cells, including monocytes, macrophages and dendritic cells, are highly metabolic and oxygen-consuming immune cells that are recruited to the inflamed foci (Glover and Colgan, 2011). The fact that transmigrating neutrophils, a subset of myeloid cells, can deplete local intestinal oxygen levels due to their ability to undergo respiratory bursts (Campbell et al., 2014), and that these cells have a unique mitochondrial-maintaining transmembrane potential via the glycerol-3-phosphate shuttle regulating aerobic glycolysis and hence energy production (van Raam et al., 2008), suggests that myeloid cells might heavily depend on HIF for metabolism and functions. Cramer et al. (2003) have demonstrated in myeloid-specific *Hif-1a* KO mice using the LysM promoter (LysM *Hif-1a* KO mice) that HIF-1α is required for macrophage energy production via glycolysis, leading to bacterial-killing abilities. Interestingly, however, Matak et al. (2015) have shown that these mice had comparable bacterial colonization in the gut when challenged with *Helicobacter* *pylori*, although they developed more severe gastritis owing to deficiency of HIF-1α in myeloid cells, impairing the production of proinflammatory molecules such as IL-6 and IL-1β. While our study was being conducted, Bäcker and et al. (2017) reported that LysM *Hif-1a* KO mice exhibit a protective phenotype from DSS-induced colitis by reduced gene expression signatures in CD11c or F4/80 myeloid cell infiltration, as well as in inflammatory cytokines such as IFN-γ, IL-17 and TNF-α analyzed in the whole colonic tissue. We, by using the hMRP8 myeloid promoter, similarly observed that hMRP8 *Hif-1a* KO mice were protected from DSS-induced colitis. However, our study revealed more in-depth lines of evidence by demonstrating that the LysM and hMRP8 promoters target different populations of cells within the myeloid lineage: LysM promoter targeted F4/80-positive colonic macrophages as well as Gr-1-positive myeloid cells, while hMRP8 largely targeted Gr-1-positive granulocytes in the colon (Fig. 2). We further extended our investigation to the role of HIF-2α in myeloid cells in DSS-induced colitis by utilizing hMRP8 *Hif-2a* KO mice. We found that HIF-2α in myeloid cells seems to play a minor role, if any, in DSS-induced colitis progression. We observed that the loss of HIF-2α in myeloid cells did not alter CD11b- or Gr-1-positive myeloid cell infiltration to the colon of mice fed with DSS (Fig. 9). Lastly, we also examined the role of HIF in myeloid cells in tumorigenesis and found that although the deletion of *Hif-1a* in myeloid cells was sufficient to protect mice from DSS-induced colitis, it was dispensable for AOM-induced tumorigenesis. It is interesting to note that Imitiyaz et al. (2010) have reported that targeted disruption of HIF-2α in myeloid cells (Bollrath et al., 2009) impairs myeloid cell migration and invasion, thereby protecting the mice from the same model of colorectal tumorigenesis. In our study, hMRP8 *Vhl* KO mice, which had an increased myeloid cell infiltration, demonstrated a rapid body weight loss and sudden mortality upon AOM treatment, likely to be due to an accelerated inflammation. However, at the time of euthanization, we found no visible tumor formation in the colon, indicating that inflammation and tumorigenesis might not always parallel. Consistent with this notion, Bollrath et al. (2009) have also reported that increased colitis induced by Stat3 deletion in enterocytes is associated with less tumorigenesis.

Although our study demonstrated that inhibition of HIF-1 in myeloid cells could be beneficial in IBD-associated colitis, other studies using epithelial-specific KO mice targeting HIF pathways suggest that induction of HIF is required to protect against intestinal inflammation. While many of those exciting HIF activators, such as DMOG, FG-4497 and AKB-4924 (Eltzschig et al., 2014), have demonstrated promising activities in intestinal inflammation models, we believe that further studies are warranted to determine spatiotemporal crosstalk between epithelium and immune cells and the role of HIF within each population.

## MATERIALS AND METHODS

### Mice

Myeloid-specific *Vhl*, *Hif-1a* and *Hif-2a* KO mice were generated as described previously (Ahn et al., 2014). In brief, mice with myeloid-specific deletion of *Vhl*, *Hif-1a* or *Hif-2a* gene were produced by Cre recombinase-mediated inactivation of VHL, HIF-1α or HIF-2α. Mice with loxP-flanking alleles in *Vhl*, *Hif-1a* and *Hif-2a* were originally created by Dr Volker Haase at Vanderbilt University (Nashville, TN, USA), Dr Randall Johnson at the University of Cambridge (Cambridge, UK) and Dr Celeste Simon at the University of Pennsylvania (Philadelphia, PA, USA), respectively, and were obtained from Dr Amato Giaccia at Stanford University (Stanford, CA, USA). They were backcrossed to C57Bl/6J purchased from the Jackson Laboratory (Bar Harbor, ME, USA) for more than seven generations. These mice were then bred to mice bearing the *Cre* recombinase gene under the human S100A8 promoter (in the background of C57Bl/6), obtained from Dr Irving Weissman at Stanford University. LysM Cre mice (stock no. 4781) and tdTomato reporter mice (ROSA-CAG-LSL-tdTomato-WPRE reporter mice; stock no. 7914) were purchased from the Jackson Laboratory. WT mice (C57Bl/6) were purchased from OrientBio (Sungnam, South Korea) and used only when we performed water versus DSS experiments. WT mice stated elsewhere in the study are littermate control mice (those not bearing the *Cre* recombinase gene) born in the same litter with myeloid-specific *Vhl*, *Hif-1a* or *Hif-2a* KO mice. Mice were maintained in a specific pathogen-free environment and had access to food and water *ad libitum*. All animal procedures were approved by the Institutional Animal Care and Use Committee (IACUC) at POSTECH. All animal experiments conformed to the relevant regulatory standards.

### DSS-induced colitis models

Colitis was induced with a modified administration techniques of Wirtz et al. (2007). Briefly, acute colitis was induced by administration of mice with 5% (w/v) DSS (MW: 36,000-50,000; MP Biomedicals, Santa Ana, CA, USA) in drinking water for 4 or 5 days. Six- to 12-week-old male mice were administered with 5% DSS in sterile distilled water for 3 days. At day 3, 5% DSS was replaced with freshly prepared 5% DSS in a new water bottle. Mice were sacrificed at day 4 or 5 (depending on the body weight loss) to harvest the colon for further analyses. Upon harvesting the colon, the colon length was measured from the cecum to the anus.

### Disease activity score evaluation for DSS-induced colitis

Disease activity score was assessed daily during 5% DSS administration to evaluate the progression of DSS-induced colitis by the following arbitrary scale: 0, no sign of toxicity; 1, looks weak; 2, body weight loss; 3, apparent bloody stool; 4, bloody anus; 5, looking severely sick and near morbidity.

### Preparation of single-cell suspension from the large intestine for FACS analysis

To prepare single-cell suspension from the lamina propria of the large intestine, the intestine was removed and placed in a Petri dish containing ice-cold phosphate buffered saline (PBS). After removing fat tissues, the intestine was opened longitudinally and cut into small pieces, followed by incubation on a magnetic stirrer at 37°C for 25 min. Tissue pieces were then filtered and collected, and subjected to enzyme digestion using collagenase D (400 Mandl units/ml, Roche Diagnostics, Risch-Rotkreuz, Switzerland) and DNase I (100 μg/ml, Biosesang, Sungnam, South Korea) at 37°C for 1 h. Digested suspensions were then centrifuged and resuspended in 70% Percoll (GE Healthcare, Little Chalfont, UK) followed by careful overlay onto 40% Percoll in order to enrich colonic mononuclear cells. This mixture was centrifuged at 805 ***g*** for 20 min at room temperature. Cells at the interphase were collected and placed in Dulbecco's modified Eagle medium (DMEM, Welgene, Gyeongsan, South Korea) containing 2% fetal bovine serum (FBS; Gibco, Carlsbad, CA, USA).

### AOM/DSS model

Eight-week-old male mice received an intraperitoneal injection of AOM (12.5 mg/kg body weight) (Sigma-Aldrich, St. Louis, MO, USA). One week later, mice were administrated drinking water containing 3% DSS for 1 week, followed by water for 2 weeks. Then, mice were administrated 3% DSS again for another week and tap water for 2 weeks thereafter.

### FACS

Single-cell suspensions were stained with the following reagents and antibodies prepared in PBS containing 1% FBS: Zombie Aqua (BioLegend, San Diego, CA, USA), PE/Cy7 anti-mouse CD11b (M1/70; eBioscience, San Diego, CA, USA) and APC-anti-mouse Ly6G/Ly6C (RB6-8C5; BioLegend). Cell populations of interest were sorted using a MoFlo (Beckman Coulter, Pasadena, CA, USA) or Astrios High-Speed Cell Sorter (Beckman Coulter). Cytometry data were analyzed by using FlowJo software 10.4.1 (Tree Star).

### mRNA fluorescence *in situ* hybridization and immunostaining

Colons were removed from mice, fixed in 4% paraformaldehyde (PFA; Daejung Chemicals, Siheung, South Korea) in PBS for 1 h at room temperature, and then incubated in 30% sucrose in PBS overnight at 4°C. Tissues were embedded in optical cutting temperature (OCT) compound (SAKURA Finetek, Torrance, CA, USA) and prepared as frozen blocks on dry ice. Frozen sections were of 8 μm thickness, and were immersed in PBS for 5 min followed by Carnoy fixative (1:3 acetic acid:methanol) for 30 min at room temperature. Slides were then washed with PBS for 5 min and incubated in proteinase K in PBS (1.5 μg/ml; Bioneer, Daejeon, South Korea) for 10 min at 37°C. After that, slides were serially dehydrated in 70%, 95% and 100% ethanol for 2 min each. Tissues were then stained by Stellaris mouse *Hif-1a* exon 2 site-specific probes (customized probe SMF-1063-5; designed by LGC Biosearch Technologies, Novato, CA, USA) along with goat anti-mouse S100A8 antibody (R&D Systems, Minneapolis, MN, USA) for 16 h at 37°C. The next day, the slides were washed with PBS three times followed by incubation with anti-goat Alexa Fluor 488 (A11055; Life Technologies, Carlsbad, CA, USA)-conjugated secondary antibodies for 45 min at room temperature. Sections were finally mounted with Prolonged mounting medium containing 4′,6-diamidino-2-phenylindole (DAPI; Life Technologies).

For myeloid cell staining, colon tissues were prepared as described above and incubated with the following antibodies overnight at 4°C where appropriate: rabbit anti-mouse CD11b (M1/70; Abcam, Cambridge, UK), FITC-conjugated Gr-1 (RB6-8C5; eBioscience), rabbit anti-mouse HIF-1α (NB100-449; Novus, Littleton, CO, USA) or rabbit anti-mouse HIF-2α (NB100-122; Novus). After washing the slides, sections were incubated with secondary antibodies, including donkey anti-goat Alexa Fluor 546 (A11056; Life Technologies), donkey anti-goat Alexa Fluor 488 (A11055; Life Technologies) or goat anti-rabbit Alexa Fluor 546 (A11071; Life Technologies)-conjugated antibodies for 1 h at room temperature, washed and counterstained with DAPI-containing mounting medium (Invitrogen, Carlsbad, CA, USA). Slides were analyzed with a Zeiss Axio Scope (Carl Zeiss, Oberkochen, Germany) with EC Plan Neofluar 10×, 20× and 40× objective lenses. Images were processed by AxioVision 4.8 software (Carl Zeiss) and quantified using Image J software (National Institutes of Health).

### Western blot analysis

Protein lysates were made from the colonic laminar propria scraping and were quantified using a Pierce BCA protein assay kit (Thermo Fisher Scientific, Waltham, MA, USA). Proteins (100 μg) were loaded onto 12% Bis-Tris SDS NuPAGE gel (Invitrogen) followed by transfer onto PVDF transfer membrane (88518; Thermo Fisher Scientific). The membrane was then incubated with primary antibodies against F4/80 (ab6640; Abcam), S100A8 (AF3059; R&D Systems), occludin (ab31721; Abcam) or claudin 5 (ab15106; Abcam) overnight, followed by incubation with secondary antibodies conjugated to horseradish peroxidase, and developed with Pierce ECL substrate (32106; Thermo Fisher Scientific) using CL-XPosure film (34091; Thermo Fisher Scientific). Actin (69100; MP Biomedicals) was used as a loading control.

### H&E staining

Upon harvesting the colon, feces were removed by flushing the colon with ice-cold PBS. The colon was embedded in OCT compound vertically from the floor of a mold and frozen immediately on dry ice. In the AOM/DSS-induced colonic tumorigenesis experiments, the colons were rolled as Swiss rolls and embedded in OCT compound. Frozen tissues were stored at −80°C until cryosectioning at 8 μm. Tissue sections were stained with Hematoxylin (Sigma-Aldrich) and Eosin (Sigma-Aldrich) and examined with a microscope (Olympus).

### TNF-α enzyme-linked immunosorbent assay

CD11b and Gr-1 double-positive cells were sorted by FACS from myeloid-specific WT or KO mice that had been fed with 5% DSS [4 days for hMRP8 *Vhl* KO (*n*=8) or WT (*n*=10) mice; 5 days for hMRP8 *Hif-1α* KO (*n*=9) or WT (*n*=10) mice]. Cells (30,000) were plated in 100 μl DMEM containing 2% FBS in 96-well plates, in triplicate, and incubated at 37°C under 5% CO_2_ for 24 h. Fifty microliters of the medium was harvested and stored immediately at −80°C until TNF-α concentration measurement with a murine TNF-α Quantikine ELISA Kit (MTA00B; R&D Systems) according to the manufacturer's instructions.

### Intestinal permeability assessment

Mice were administrated using oral gavage with FITC-dextran (4 mg/10 g body weight; Sigma-Aldrich) and blood samples were collected through retro-orbital bleeding at 4 h after the oral administration. Plasma samples were prepared by centrifugation of the blood samples at 1200 ***g*** for 10 min. FITC was measured in 50 μl plasma by using a plate reader infinite F200 PRO (TECAN, Männedorf, Switzerland) at 488 nm. FITC-dextran prepared in saline was used to generate a standard curve.

### Statistical analysis

Data were analyzed by Student’s *t*-test, or one- or two-way ANOVA, using GraphPad Prism version 4 software (San Diego, CA, USA). *P*-values less than 0.05 were considered statistically significant.
